# Soil Respiration in Semiarid Temperate Grasslands under Various Land Management

**DOI:** 10.1371/journal.pone.0147987

**Published:** 2016-01-25

**Authors:** Zhen Wang, Lei Ji, Xiangyang Hou, Michael P. Schellenberg

**Affiliations:** 1 Grassland Research Institute, Chinese Academy of Agricultural Sciences, Hohhot, 010010, China; 2 Semiarid Prairie Agricultural Research Center (SPARC), AAFC-AAC, Box 1030, Swift Current, Saskatchewan, S9H 3X2, Canada; Beijing Normal University, CHINA

## Abstract

Soil respiration, a major component of the global carbon cycle, is significantly influenced by land management practices. Grasslands are potentially a major sink for carbon, but can also be a source. Here, we investigated the potential effect of land management (grazing, clipping, and ungrazed enclosures) on soil respiration in the semiarid grassland of northern China. Our results showed the mean soil respiration was significantly higher under enclosures (2.17μmol.m^−2^.s^−1^) and clipping (2.06μmol.m^−2^.s^−1^) than under grazing (1.65μmol.m^−2^.s^−1^) over the three growing seasons. The high rates of soil respiration under enclosure and clipping were associated with the higher belowground net primary productivity (BNPP). Our analyses indicated that soil respiration was primarily related to BNPP under grazing, to soil water content under clipping. Using structural equation models, we found that soil water content, aboveground net primary productivity (ANPP) and BNPP regulated soil respiration, with soil water content as the predominant factor. Our findings highlight that management-induced changes in abiotic (soil temperature and soil water content) and biotic (ANPP and BNPP) factors regulate soil respiration in the semiarid temperate grassland of northern China.

## Introduction

Soil respiration is the second largest carbon (C) flux between terrestrial ecosystems and the atmosphere in the global C cycle [1,2], and plays an important role in regulating the soil carbon pool and ecosystem C-cycling [3,4]. Soil respiration is the dominant influence on the carbon dioxide (CO_2_) released from the soil surface, generated mainly from a combination of the metabolic activity of roots and microorganisms [5]. Many studies were conducted to examine the effect of climate change factors on soil respiration, such as increasing temperature [6] and atmospheric CO_2_ concentration [7], and changes in precipitation patterns [8]. Land management changes are the most dominant component of global climate change in terms of their impacts on terrestrial ecosystems [9,10], as they profoundly alter land cover [11, 12] and biogeochemical cycles [13,14,15]. Numerous studies show that changes in land management alter soil respiration, but the sign and magnitude of land management effects on soil respiration are highly uncertain [10, 11, 16, 17].

Grazing and mowing are two major grassland management practices used in the semiarid temperate steppe region of northern China, while the enclosure of grassland is widely used in the region for grassland rehabilitation and to increase grass yields for hay cuts. These management measures have been shown to change soil respiration by different mechanisms [5, 10]. Livestock preference for different species of forage and trampling of soil and deposition of dung and urine alter the plant community structure [18] and production [12], as well as the size and composition of carbon and nutrient pools [19], soil microorganism communities, and physical and chemical properties of soil [20]. All of these factors have some influence on soil respiration. Long-term livestock grazing, especially overgrazing, is one of the major causes of soil and vegetation degradation in grassland environments [12, 18], which potentially affects soil respiration by indirectly altering vegetation and soil physicochemical properties [19, 20]. Grazing was shown to decrease the annual soil respiration by 33% in an alpine meadow on the Tibetan Plateau [10], but grazing increased soil respiration in a shortgrass steppe in Colorado [17]. Clipping has the potential to considerably change soil respiration because it alters the microclimate. The amount of aboveground litter is low under clipping, which breaks down the inherent cycling between aboveground and belowground plant organs, and simulates root growth through the removal of aboveground plant biomass [21]. Varying results have been shown in previous studies into the effect of clipping on soil respiration: decrease [22], little change [23] or no change [24]. Enclosure, as a method of restoring grassland, has a major effect on soil respiration because it changes plant diversity, community structure and productivity by excluding livestock [1], more carbon and nutrients are retained in aboveground plant material. The differences among these researches partially arise from the lack of mechanistic understanding of the feedback of soil respiration to land management. Therefore, further research is needed to investigate the mechanisms behind the influence of land management on the terrestrial C cycle.

Grasslands cover approximately 40% of the global terrestrial ice-free surface, with the Eurasian grassland ecosystems in the semiarid temperate regions being the largest. Grasslands occupy 400m ha of the land surface in China [25]. Grasslands are sensitive to land management practices that alter C and N inventories [7, 23]. Changes in the net C balance of grassland ecosystems may therefore have significant implications for regional and global C balance. However, their contribution to local and regional soil respiration is still unclear under different land management. To address this knowledge gap, a three-year field experiment was conducted to examine the potential impacts of different land management practices (grazing, clipping and ungrazed enclosures) on soil respiration in a semiarid temperate steppe in northern China. Under changes of climate and precipitation patterns, the effects of land management on seasonal and interannual soil respiration may interact with the precipitation, which fluctuates across years in semiarid temperate grasslands [2]. The objective of current study was to understand 1) the causal relations and pathways of the effects of grazing and clipping on soil respiration; and 2) the interactive effects of climate (temperature and precipitation) and land management (grazing, clipping and enclosure) changes on soil respiration, and their relations with plant production.

## Material and Methods

### Study site

**Fig 1:**
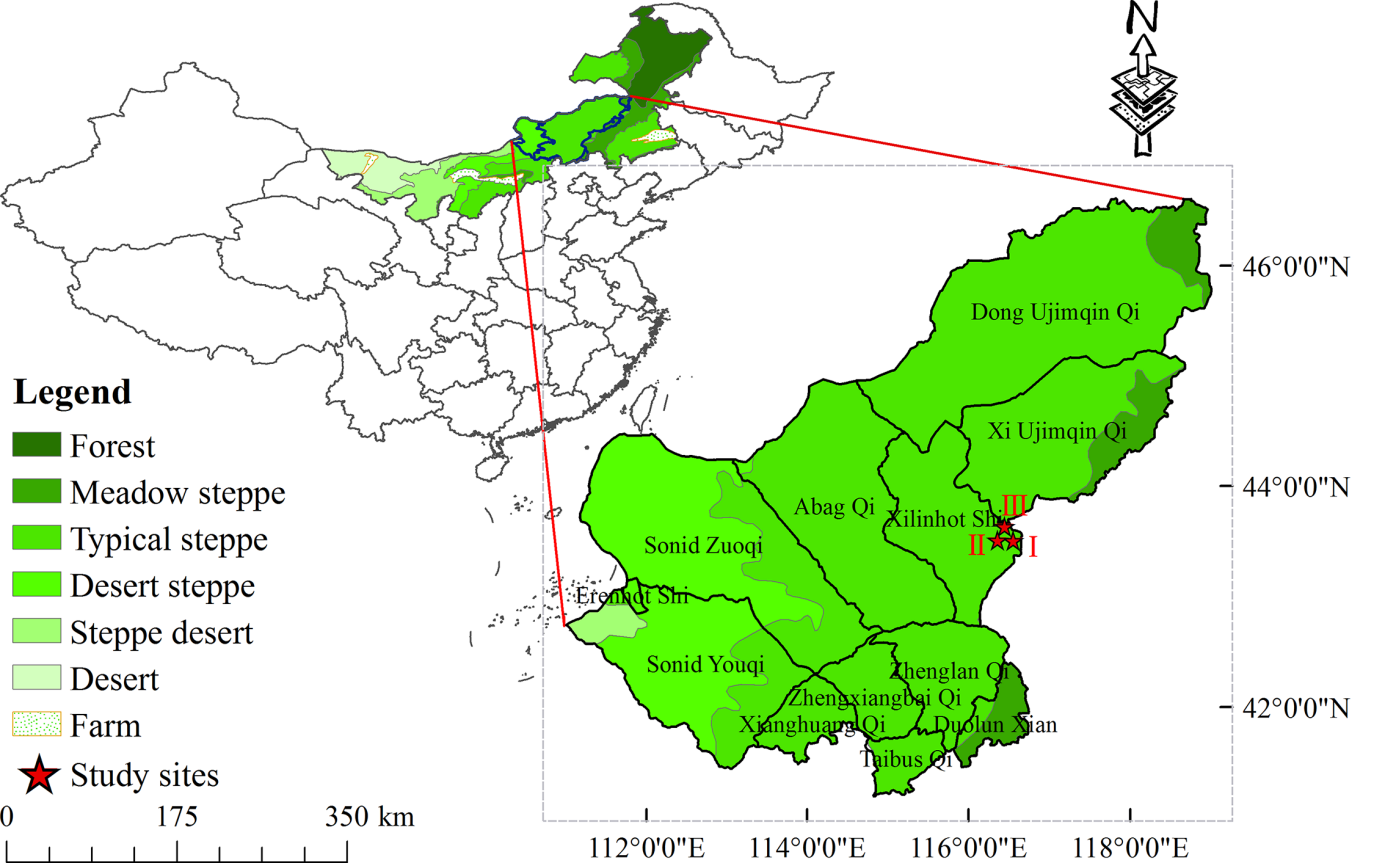
Location of the study area and experimental sites.

The experiment was conducted at the Inner Mongolia Grassland Ecosystem Research Station (IMGERS), located in the Xilin River Basin (Site I-III: 43°30′-44°19′,116°12′-116°72′), Inner Mongolia, P. R. China, which was a typical steppe zone with a semiarid continental temperate steppe climate (Fig 1).

The mean annual air temperature is 0.4°C with the lowest mean monthly temperature occurring in January (−21.4°C) and the highest mean monthly temperature in July (18.0°C). The region is dry in spring and humid in summer with a long-term mean annual precipitation (MAP) of 278.7 mm (1953–2009) and with more than 70% of rainfall occurring from May to August. The soils are classified as Calcic Chernozems (IUSS Working Group WRB 2006) [26] or loamy sand based on texture, with similar physiochemical properties [27]. Typical steppe vegetation at the site is dominated by the C_3_ grasses *Leymus chinensis*(Trin.) Tszvelev, *Stipa grandis* Griseb., and C_4_ grasses C*leistogenes polyphylla* Keng., and semi-shrub *Artemisia frigida* Willd.

### Experimental design

The experimental design consisted of three land management practices: grazed (G), clipping (C), and enclosure (E) at three typical steppe sites (Sites I–III). The total number of plots was nine and the area of each plot was 4.5 ha. Free-range seasonal grazing was adopted in the study area from 1950–1979, but the sheep population then increased dramatically from 1979 in the semiarid temperate grassland when land reform was implemented along with the household responsibility system in China. The grazing study site has been grazed since 1979 and is dominated by *Stipa grandis*, *Artemisia frigida* and *Cleistogenes polyphylla*. The plants in the clipping plot have been clipped twice each year (early May and late August) since 1998 (no grazing). This study site was dominated by *Leymus chinensis* and *Stipa grandis*. The ungrazed enclosures were established relatively recently, 10 years ago, at the three study sites on grassland that had previously been moderately grazed and had maintained primary production, biodiversity and habitat structure. The dominant species in the enclosures were *Leymus chinensis* and *Stipa grandis*. The nine plots were located in a relatively small area with similar climate conditions, such as air temperature and precipitation.

### Field measurement

Soil respiration was measured twice each month, from late May to early October, using a Li-Cor 8100 IRGA (Li-Cor, Lincoln, NE, USA). PVC collars (11 cm in diameter and 5 cm in height) were permanently inserted 2–3 cm into the soil to measure soil respiration at each plot. Every plot had 5 PVC collars. Living plants inside the soil collars were removed at the soil surface before we measured the soil respiration to eliminate aboveground plant respiration. The soil respiration chamber was placed on the PVC collars, scrubbing the CO_2_ to ambient levels, and determining soil CO_2_ efflux over a short period. The Li-Cor 8100 automatically recorded the data at 5-s intervals. Each measurement took approximately 3 min to complete. The bi-monthly soil respiration measurements (the diurnal rate of soil respiration) were recorded (2-h intervals) on clear, sunny days from 2010 to 2012. The soil temperature and soil water content were also determined with the soil respiration rate. The soil temperature measurement was made at a depth of 10 cm, adjacent to each PVC collar by a thermocouple probe connected to the Li-8100, while soil gravimetric water content was determined monthly from May to October by sampling soil from 0–10 cm, using a soil core 3.5 cm in diameter. Ten soil cores were extracted at each plot. The weight loss, after drying at 105°C for 24 h, was used to calculate soil moisture.

Aboveground net primary production (ANPP) was determined in August 2010–2012 by harvesting ten 1-m^2^ quadrats inside an enclosure plot. For the other two land managements, ten 1-m^2^ quadrats were harvested in 10 portable cages (1.5 m × 1.5 m) that were established in each clipped (before early May) and grazing (before grazing began) plot in the spring. All aboveground plant materials were cut to the ground surface (including living aboveground biomass, standing litter, and ground litter) in the quadrat (1 m × 1 m). We separated plant aboveground tissue (living aboveground biomass and standing litter) from standing litter of the previous year and ground litter, and into different species. Ten 1-m^2^ quadrats were harvested in every plot (sampling locations were spaced approximately 20 m apart) for a total of 90 in nine plots. Harvested biomass was oven-dried at 65°C for 48 h and then weighed. ANPP was calculated as the sum of aboveground biomass for all plant species.

Belowground net primary production (BNPP) was measured in each plot using the ingrowth core method [28]. Ten 50-cm-deep cylindrical holes were excavated using a soil auger (7 cm in diameter) at the same sites from which ANPP was obtained in each experimental plot in early May from 2010 to 2012. Ten root meshes (1 mm) were set in each plot (sampling locations were spaced approximately 20 m apart) by transect, with a total of 90 in nine plots. Root mesh cores (7 cm in diameter and 50 cm in length) were placed into each hole. The soil was then returned to its original hole with the root meshes after removing root material greater than 2 mm using sieves. In late October, we collected the root growth samples by taking out the root mesh from each hole. The dry root mass was oven dried at 65°C for 48 h and weighed.

### Data analysis

Repeated-measures analysis of variances (RMANOVAs) was used to examine the interannual variability of soil temperature, soil water content, ANPP, BNPP and soil respiration over the growing season based on land management practices for 2010–2012. One-way ANOVA was used to examine the statistical difference in seasonal averages for soil temperature, soil gravimetric water content, ANPP and BNPP between land managements. Structural equation models (SEMs) were used to analyses hypotheses that may explain the pathways responsible for the effects of land management on soil respiration influenced by soil temperature, soil gravimetric water content, ANPP and BNPP [29]. Regression, including the correction and stepwise multiple linear analyses, was used to examine the relationships of soil respiration with soil temperature, soil gravimetric water content, ANPP and BNPP. All statistical analyses were conducted with SAS software (SAS Institute Inc., 2010, Cary, NC, USA).

## Results

### Soil microclimate

**Fig 2:**
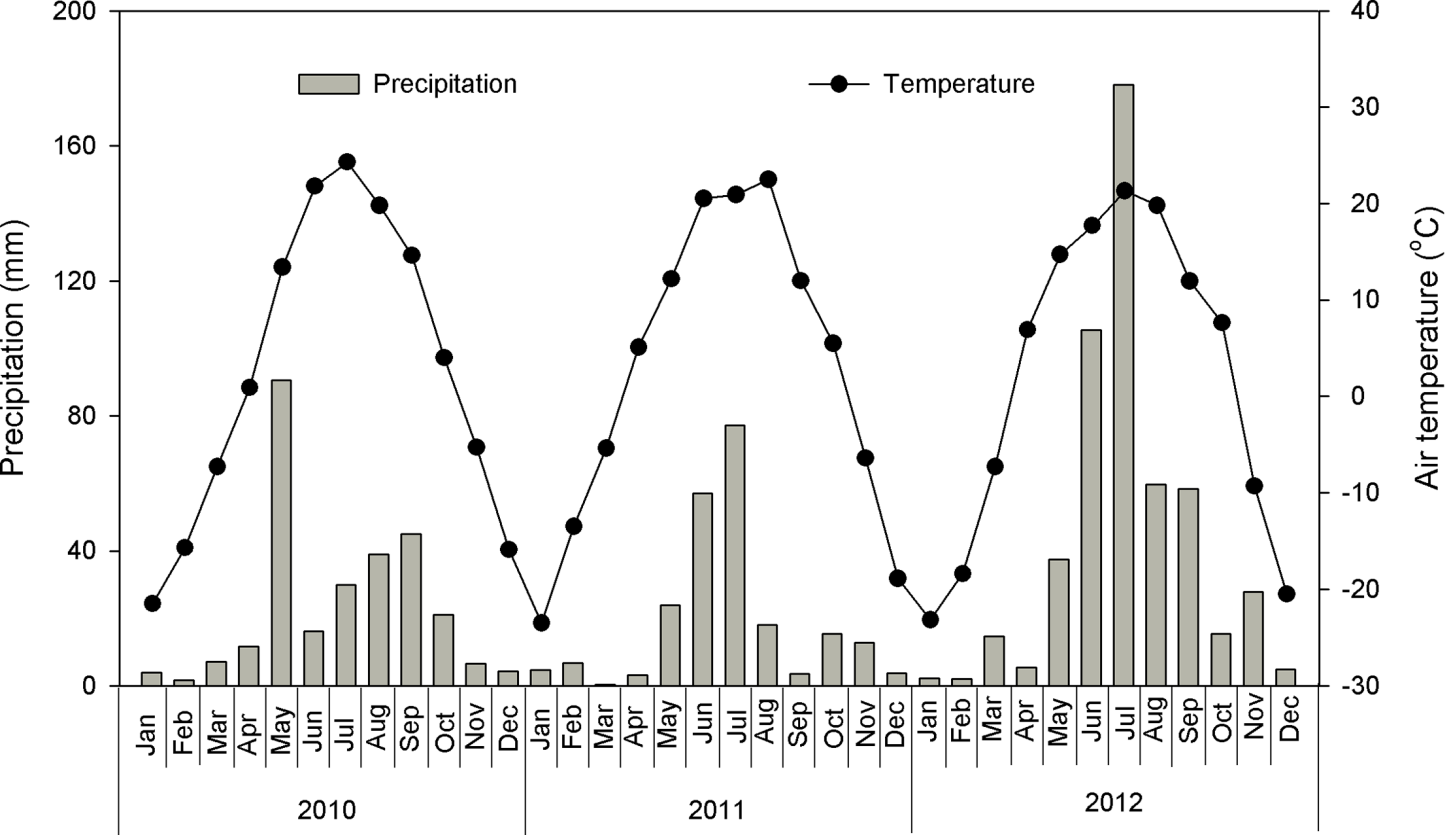
Monthly precipitation (bars) and monthly mean air temperature (line) for 2010–2012.

The annual precipitation in 2010 (276.9 mm) was close to the long-term average (278.7 mm), lower in 2011 (226.7 mm) and considerably higher in 2012 (511.7 mm, Fig 2). Soil temperature was the highest under enclosure comparing to grazing or clipping treatments (*P*<0.001, repeated ANOVA, Table 1). The gravimetric water content varied significantly (*P*< 0.001) by year with values highest in 2012 and lowest in 2011 (Table 1).

**Table 1:** Response of soil temperature during the plant growing season (T), soil gravimetric water content (SW), aboveground primary productivity (ANPP), belowground primary productivity (BNPP) and soil respiration (Rs) to grassland management practices over 3 years in the semiarid grassland.

		T	SW	ANPP	BNPP	Rs
		°C	%	-----g.m^−2^------		μmol.m^−2^.s^−1^
Treatment	Grazing	11.14B	10.63B	243.33B	1095C	1.65B
	Clipping	10.54C	11.03B	251.93B	1294B	2.06A
	Enclosure	12.14A	12.05A	300.27A	1343A	2.17A
Year	2010	10.94B	10.69B	273.88B	1045B	1.89B
	2011	11.82A	9.00C	196.76C	991C	1.42C
	2012	11.05B	14.02A	324.90A	1697A	2.57A
ANOVA				*P* > F		
	Treatment	< .0001	< .0001	< .0001	< .0001	< .0001
	Year	< .0001	< .0001	< .0001	< .0001	< .0001
	Treatment×Year	< .0001	0.002	0.484	< .0001	0.119

### The effect of grazing, clipping, and enclosure on plant community productivity

**Fig 3:**
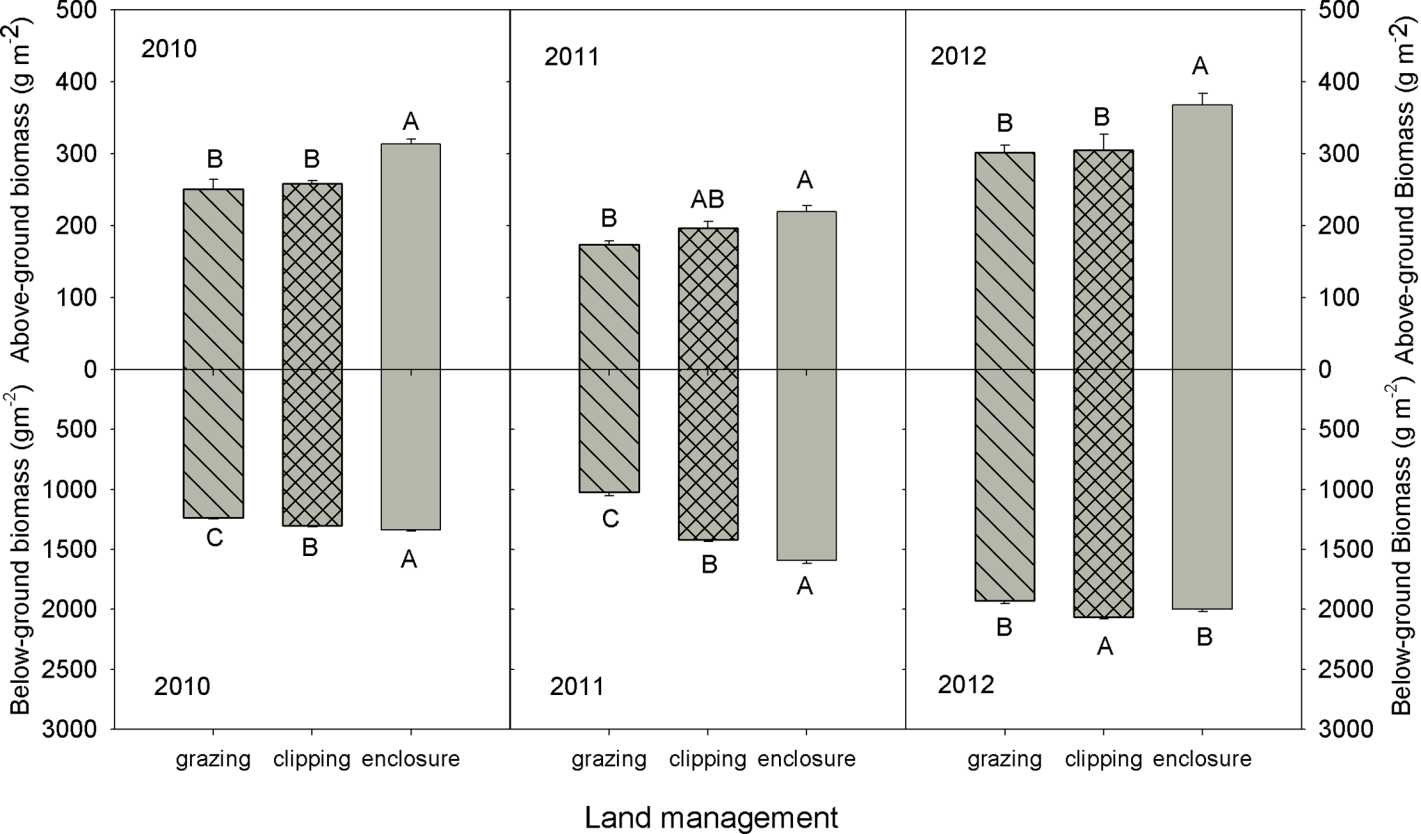
Effects of grazing, clipping, and enclosure on aboveground net primary productivity (ANPP) and belowground net primary productivity (BNPP) 2010–2012 (inset mean ± SE).

ANPP was 19.0% and 16.1% less in grazing and clipping treatments, respectively, relative to that of the enclosure treatment (*P* < 0.05, Table 1). The ANPP was the highest under the enclosure treatment in every year (*P*< 0.05, Fig 3). The BNPP was significantly different (*P*< 0.001) between the treatments over the 3 years: enclosure (1343 g.m^-2^) > clipping (1294 g.m^-2^) > grazing (1095 g.m^-2^) (Table 1). In addition, the BNPP was greatest under the enclosure in both 2010 and 2011 (*P*< 0.05, Fig 3), but under clipping in 2012 (Fig 3).

### The effect of grazing, clipping, and enclosure on soil respiration

**Fig 4:**
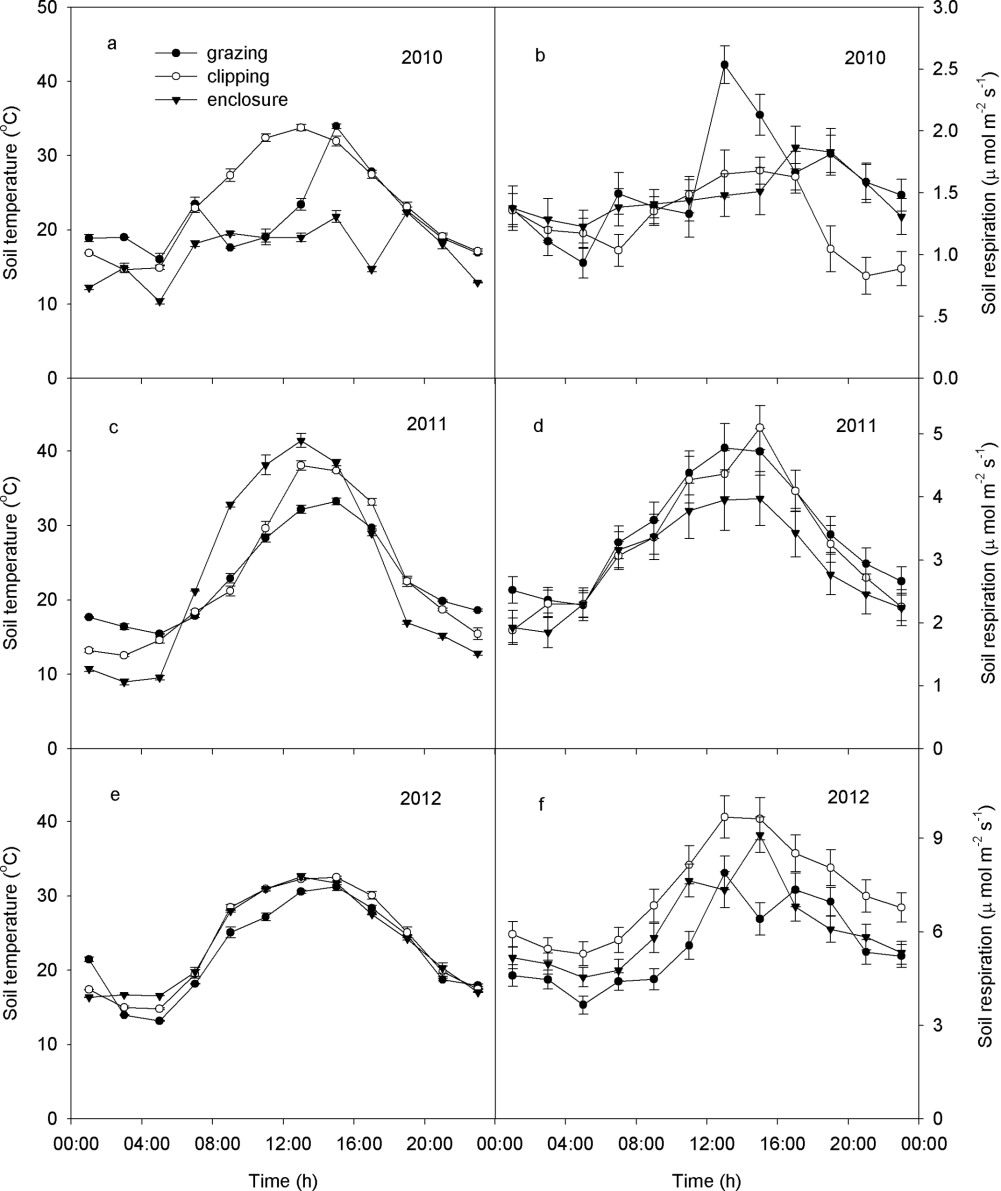
Diurnal variations in soil temperature (a, c, e) and soil respiration (b, d, f) under grazing, clipping, and enclosure on August 6–7, 2010, 2011 and 2012 (inset mean ± SE).

A significant diurnal difference was detected on soil temperature and soil respiration (time effect, *P* < 0.001) as shown by data from August each year (Fig 4A–4F). The peak temperatures occurred between 14:00 and 17:00 in the different land managements, while soil respiration reached a maximum at 12:00–15:00 (Fig 4A–4F).

**Fig 5:**
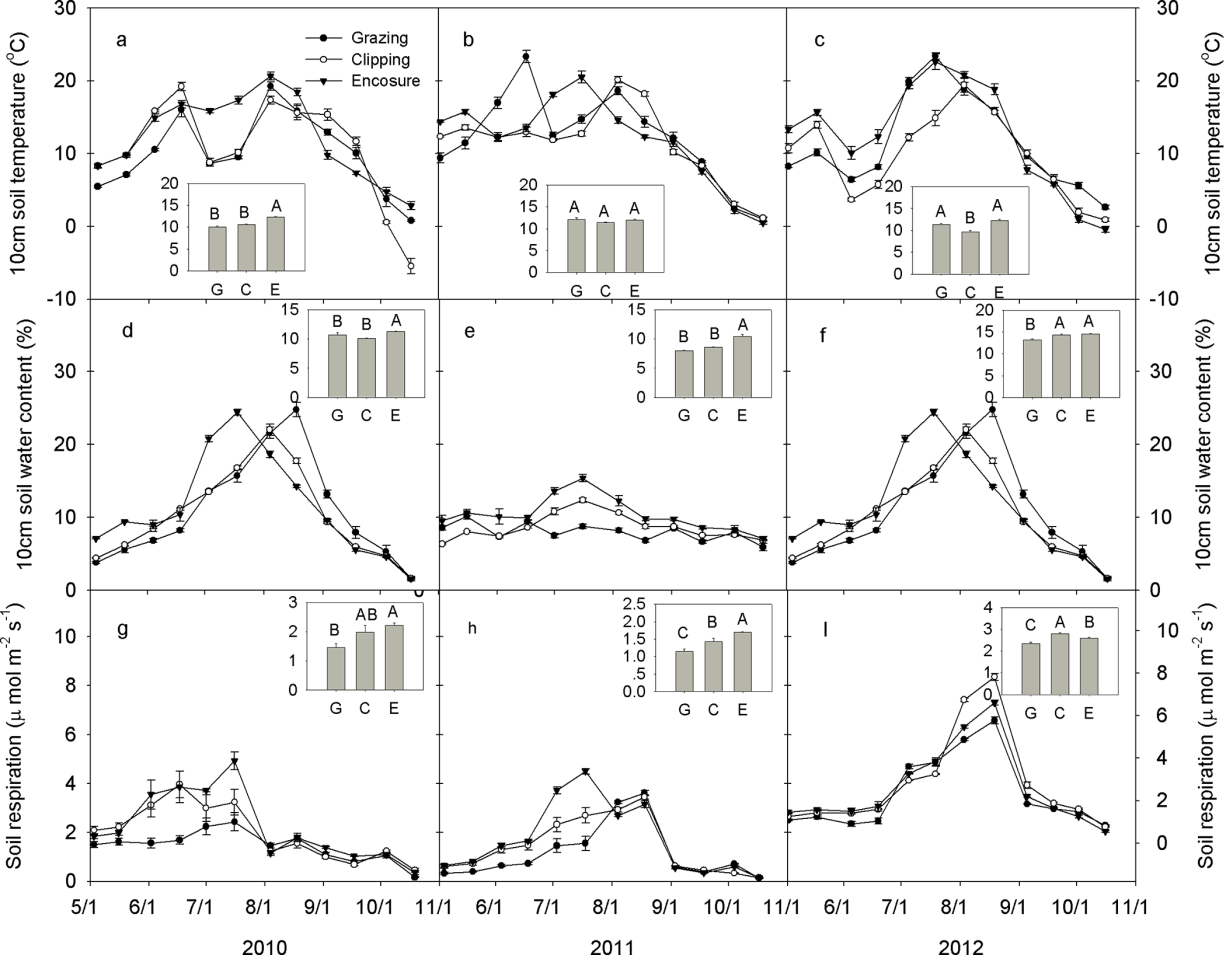
Seasonal dynamics and mean (inset mean ± SE) of soil temperature (a, b, c), soil gravimetric water content (d, e, f), and soil respiration (g, h, i) for 2010–2012. G: grazing, C: clipping, E: enclosure.

A significant difference in soil respiration rates was detected among grazing, clipping, and enclosures over the 3 years (*P* < 0.001, Table 1). Compared with the ungrazed enclosure, soil respiration was significantly reduced by grazing (*P*< 0.001, Table 1). The soil respiration was highest under the enclosure in both 2010 (2.22 μmol. m^−2^.s^−1^, Fig 5G) and in 2011 (1.69 μmol. m^−2^.s^−1^, Fig 4H), but highest in the clipping treatment for 2012 (2.78 μmol. m^−2^.s^−1^, Fig 5I).

### The effect of biotic and abiotic factors on soil respiration

**Fig 6:**
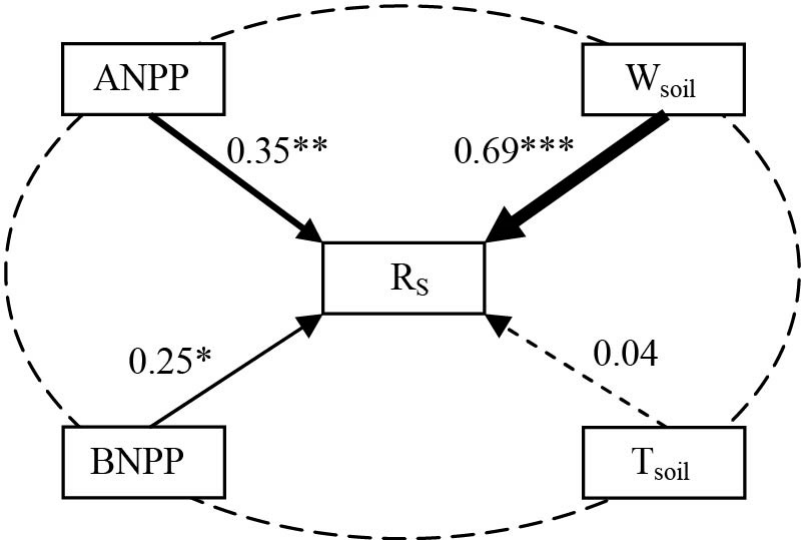
Path analysis on the impacts of land management changes on soil respiration through affecting abiotic and biotic factors. Solid and dashed arrows represent significant (*P*< 0.05, marked *; *P*< 0.01, marked **; *P* < 0.001, marked *** in the figure) and non-significant (*P*> 0.05) paths. Values associated with arrows represent standardized path coefficients. T_air_: soil temperature; W_soil_: soil water content; ANPP: aboveground net primary production; BNPP: belowground net primary production; R_s_: soil respiration.

**Fig 7:**
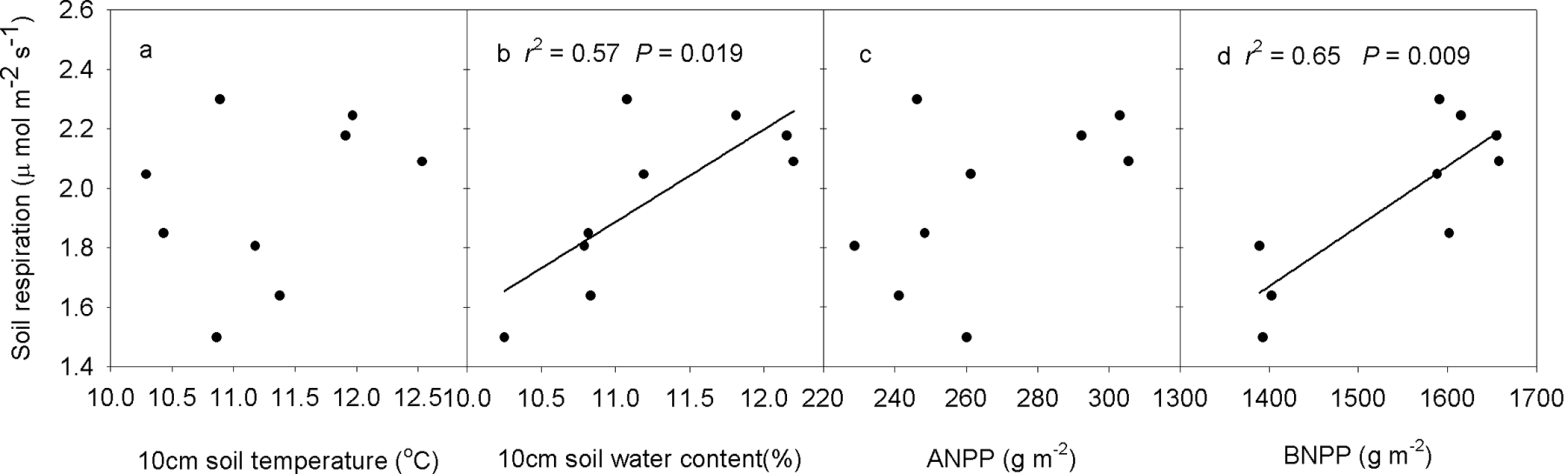
Temporal dependence of soil respiration on soil temperature (a), soil water content (b), ANPP (c) and BNPP (d) across the three growing seasons.

SEMs adequately fitted the data, with interactive networks of environmental factors and production regulating soil respiration (Fig 6). Soil water content (*P*< 0.001), ANPP (*P*< 0.01) and BNPP (*P*< 0.05) regulate soil respiration, and soil water content is the most important contributing factor determining the change in soil respiration (Fig 6). Soil respiration increased linearly with increasing soil water content and BNPP (Fig 7B and 7D). BNPP alone explained 68.9% of the seasonal variation in soil respiration based on stepwise multiple regression analyses.

**Fig 8:**
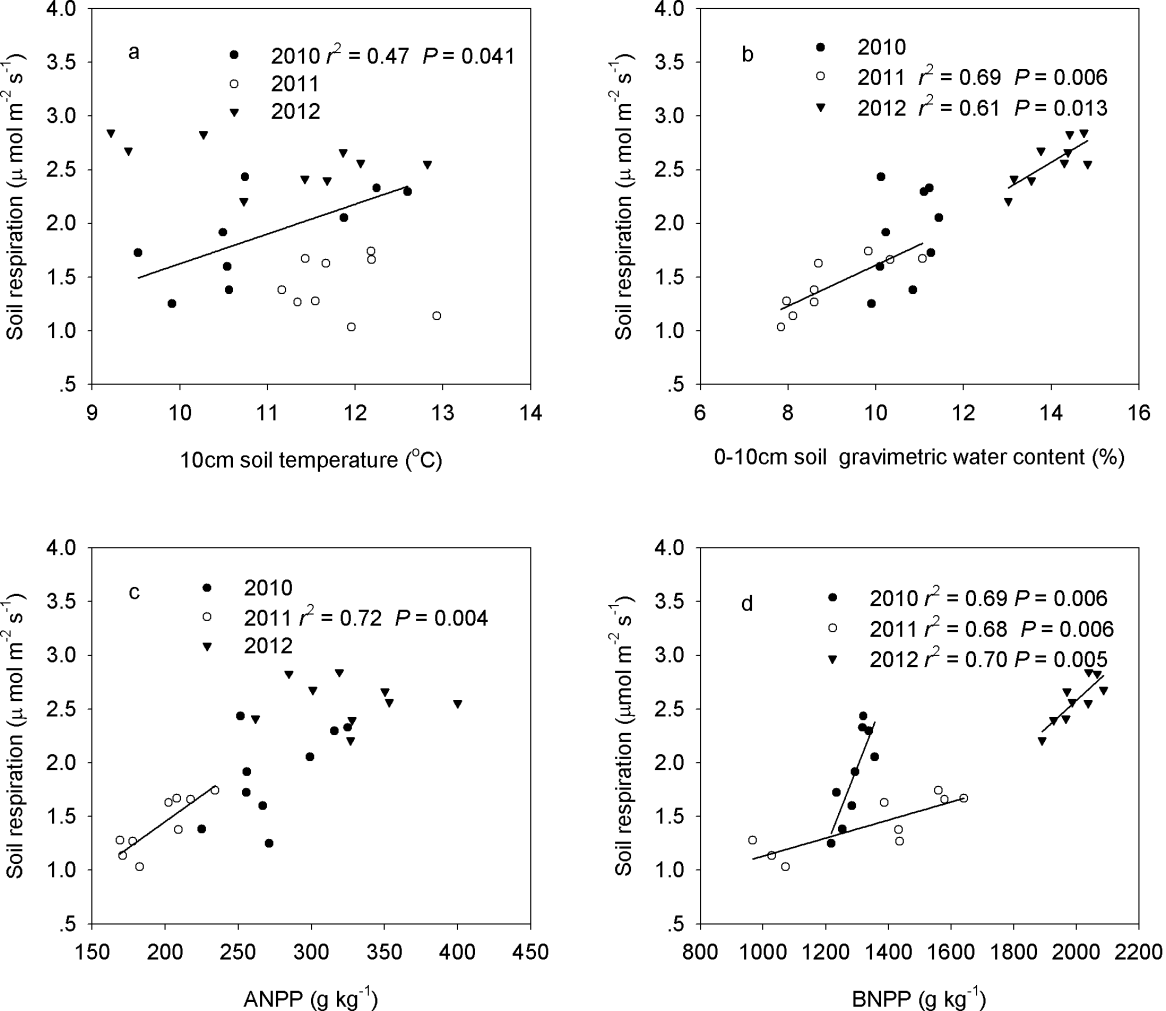
Spatial dependence of seasonal mean soil respiration on soil temperature (a), soil water content (b), ANPP (c), and BNPP (d), across nine plots for 2010–2012.

Across the nine plots, seasonal mean soil respiration displayed a positive linear correlation with soil temperature in 10cm soil layer (*P* = 0.041, Fig 8A) and BNPP (*P* = 0.006, Fig 8D) in 2010. Stepwise multiple regression analyses demonstrated that 68.8% of the variation in soil respiration could be attributed to BNPP (*P* = 0.006) in 2010. In 2011, seasonal mean soil respiration displayed a positive linear dependence on 0–10 cm soil gravimetric water content (*P* = 0.006, Fig 8B), ANPP (*P* = 0.004, Fig 8C) and BNPP (*P* = 0.006, Fig 8D). Stepwise multiple regression analyses demonstrated that ANPP contributed to 71.4% of the variation in soil respiration (*P* = 0.004). For the seasonal mean soil respiration in 2012, a positive linear relationship was detected between soil respiration and 0–10 cm soil gravimetric water content (*P* = 0.013), and between soil respiration and BNPP (*P* = 0.005, Fig 8D). BNPP alone was responsible for 69.9% (*P* = 0.005) of the variation in soil respiration.

**Fig 9:**
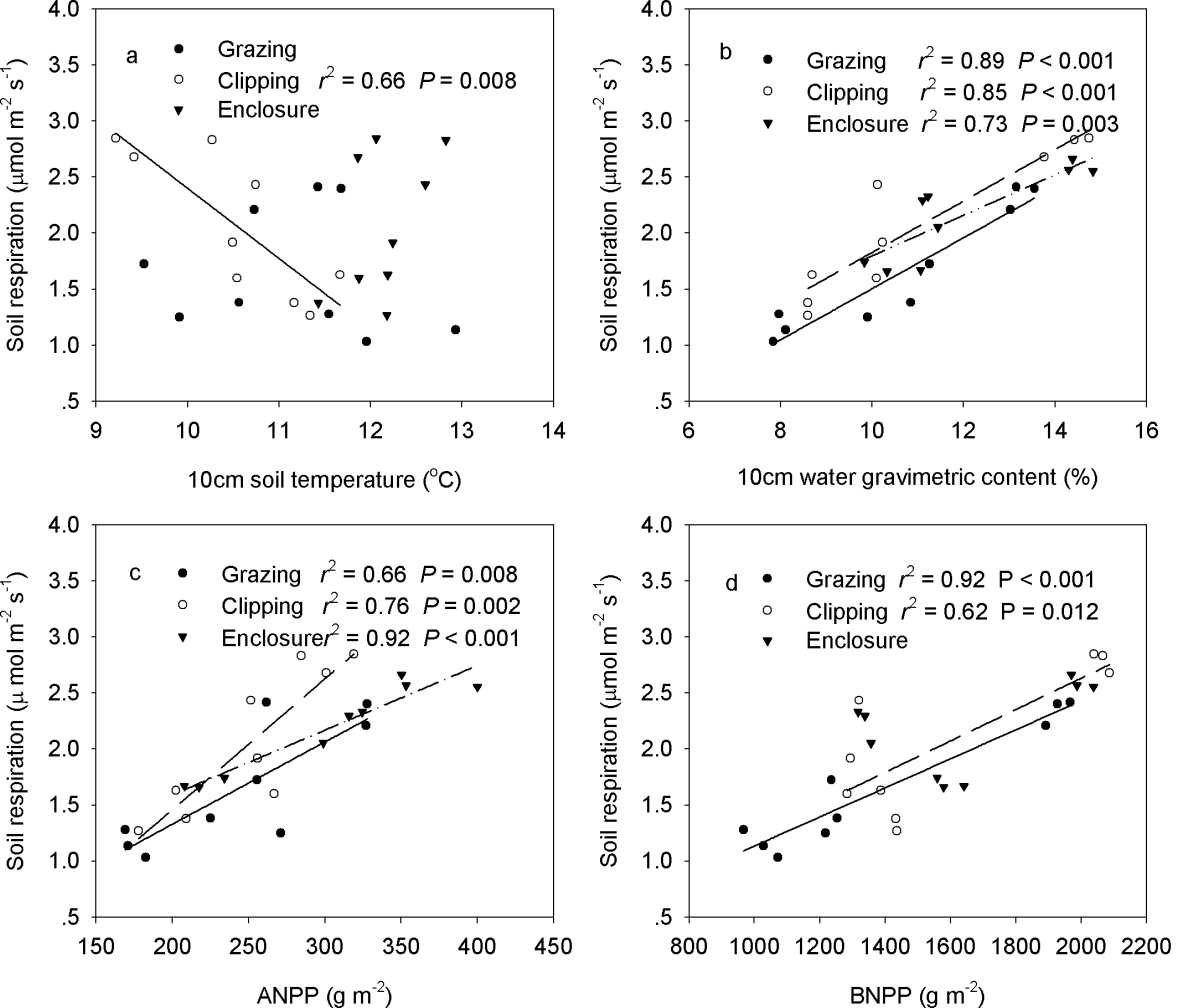
Spatial dependence of seasonal mean soil respiration of grazing, clipping, and enclosure on 10-cm soil temperature (a), 0–10-cm soil water content (b), ANPP (c), and BNPP (d), across nine plots.

Across the three growing seasons, soil respiration decreased linearly with soil temperature in the clipped treatment (*P* = 0.008, Fig 9A). Soil respiration increased linearly with increasing soil water content in all the grazed (*P*< 0.001), clipping (*P*< 0.001), and enclosure (*P* = 0.003) plots (Fig 9B). Similar to soil water content, soil respiration increased linearly with increasing ANPP because of enclosure (*P*< 0.001), clipping (*P* = 0.002) and grazing (*P* = 0.008) (Fig 9C). Soil respiration increased linearly with BNPP for grazing and clipping, with a steeper slope of regression for grazing (*P*< 0.001) than clipping (*P* = 0.012) (Fig 9D). BNPP alone explained 91.9% of the seasonal variation in soil respiration under grazing based on stepwise multiple regression analyses. Soil water content contributed 84.9% (*P*< 0.001) of the spatial variation of clipping. Stepwise multiple regression analyses showed that ANPP (partial *R*^2^ = 92.0%, *P*< 0.001) and soil temperature (partial *R*^2^ = 5.4%, *P* = 0.012) together accounted for 97.4% of the spatial variation in the enclosure treatment over the 3 years.

## Discussion

### Soil respiration in semiarid temperate steppe

Soil respiration is lower in the temperate steppe of northern China than that reported for alpine meadows [30], but similar to that in other semiarid ecosystems [8, 31, 32]. The diurnal variation of soil respiration is expected to be mainly affected by the diurnal change in soil temperature over the 3 years (Fig 4A–4F), which is in agreement with results from the Northern Great Plains of North America [16]. Though, the potential soil respiration rate on any day would be driven by the soil water content (Fig 6). The temporal dynamics of soil respiration followed the seasonal patterns of soil water content under all three land management practices in this experiment (higher in summer and lower in spring and autumn (Fig 5D–5I). High soil water content enhances plant growth. Plants allocate more C to belowground parts, as a survival strategy, to maximize their capacity to take up water and nutrients in water-limited ecosystems [33]; subsequently, it provides more C substrates for the activities and respiration of roots and microorganisms [4, 34], thus regulates soil respiration. The BNPP was significantly greater than ANPP indicating that plants were devoting more resources to roots. In this environment that would aid survival through nine cold months from autumn, through spring. SEMs were able to identify the soil water content as the predominant factor driving soil respiration in the studied ecosystems (Fig 6). This result is also supported by the positive linear correlations between soil respiration and soil water content at the temporal scale (Fig 7).

### Effects of different land management practices on soil respiration

The grassland enclosure excluded the effects of animal grazing on plant production, resulting in a higher soil respiration rates. In addition, the higher soil respiration under enclosure protection, compared to that under grazing (Table 1), might be attributed to the difference in species composition of plant communities, which influence soil respiration by altering the quality of detrital inputs into soil [35]. In our study, the grazing preference for dominant species (e.g. *Leymus chinensis*, with high nitrogen content) was related to the changes in plant communities [36, 37]. Livestock prefers to graze plant species of high nitrogen content, whereas species containing high nitrogen content is able to grow well in the enclosures. Therefore, high nitrogen (N) concentration in plant tissue resulted in high respiration rates because of the exclusion of the grazing effect [38, 39].

Relative to the enclosure, the reduction (31.5%) in soil respiration by grazing over the 3 years in this semiarid temperate grassland is consistent with reports from other grassland studies [5, 31]. Low soil respiration in grazing may be mainly due to low soil water content, leading to low ANPP and BNPP and a reduction in decomposition by microorganisms [10]. In addition, grazing-induced vegetation fragmentation is known to cause a reduction in soil respiration [40]. In semiarid environments, vegetation patches (especially the larger patches) provide favorable habitats for maintaining species richness, improving seedling establishment and increasing community productivity [41]. However, the break-up of vegetation patches into smaller units under grazing results in vegetation fragmentation. This process negatively affects plant reproduction [42] and increases the risk of plant species loss [43]. Vegetation fragmentation, the decrease of ANPP and BNPP under grazing, and the positive relationship between these factors and soil respiration (Fig 9), suggests a decrease in soil respiration under grazing.

Our finding that clipping causes no change in soil respiration (Table 1) is inconsistent with the increase in soil respiration caused by increasing soil temperature in temperate mountain grassland reported in a previous study [11]. Our results could be explained by lower soil respiration in clipping than in enclosure in 2011 (Fig 5I), higher soil respiration in clipping than in enclosure in 2012 (Fig 5J), and no difference in 2010 (Fig 5G), which led to no overall change in soil respiration under clipping, compared to that in enclosure plot. In addition, annual clipping has little impact on soil respiration because the removal of aboveground biomass is counteracted by more C allocation belowground (Fig 3). Root respiration is only slightly impacted because carbohydrate reserves sustains root metabolism. Furthermore, microbial respiration responds strongly to short-term changes in assimilate supply by clipping over a short time period [44], which explains why no change occur in soil respiration under clipping. Higher soil respiration under clipping than grazing in our study may be attributed to the significant decrease in BNPP under clipping than grazing (Fig 3). Our results suggest that land management has profound impact on terrestrial ecosystems by altering soil respiration, which are related with plant community productivity and biogeochemical processes [45].

### The effect of biotic and abiotic factors on soil respiration

Grazing significantly reduces the soil respiration rate throughout the plant growing season (May-October) compared with the enclosure (Table 1; Fig 5G–5I), which is consistent with a relatively long-term experiment in a high grazing-intensity grassland ecosystem in East Asia [10]. However, the observation from the present study is contrary to earlier research showing that grazing induced a higher CO_2_ efflux in a tallgrass prairie [16]. Low soil respiration in grazing may be mainly due to low soil water content (Table 1), and low ANPP and BNPP in our case (Fig 3).

The effect of land management on soil respiration varied over the three growing seasons in our studied ecosystem. In 2010, because of precipitation being similar to the long-term MAP, temperature played an important role in determining the net primary productivity and microbial activity under various land management examined. The positive correlations among soil respiration, soil temperature and BNPP (Fig 8A and 8D) support the hypothesis that soil temperature regulates spatial variation of soil respiration in the semiarid grassland of northern China [31]. In the dry growing season of 2011, soil water content was the prevailing control factor on soil respiration during the plant growing season [46]. Because of the low precipitation during June–July 2011 (Fig 2), soil water content was the primary limiting factor for net primary productivity [47] and microbial activity [34]. Lower soil water content in the plant peak-growing period (August) in grazing compared with ungrazed enclosure plots could have resulted in slow plant growth, thus lower ANPP, BNPP (Fig 3), which in turn resulted in low root and microorganism respiration. Therefore, the lower soil water content in the grazing plots limited soil respiration. The soil gravimetric water content, ANPP and BNPP were the determining factors for high soil respiration within the enclosure for 2011 (Fig 8B–8D). In the 2012 growing season, precipitation was relatively abundant over the entire plant growing season (Fig 2), and the NPP was the main limiting factor for soil respiration (Fig 3). The high BNPP increased root respiration, resulting in a higher respiration in clipping plots. In addition, the stepwise multiple regression analyses showed that the change of soil respiration could be attributed to BNPP in 2010 and 2012 (2010: 68.8% and 2012: 69.9%). Our results are in agreement with a previous study in the forest ecosystem [9], in which the root respiration contributes to 60.4% of total soil respiration. These results are also in agreement with findings from other ecosystems [23, 48]. Our results highlight the importance of soil temperature, soil water content and plant community production (above and below) in regulating the responses of soil respiration to grazing, clipping, and enclosure.

### Roles of temporal variation in precipitation

The timing of precipitation is crucial to determine the seasonal soil respiration in the studied temperate steppe, and this result is consistent with those widely observed in other temperate semiarid ecosystems [24]. The seasonal soil respiration between land managements could largely be explained by the variation in seasonal precipitation. In the 2011 dry growing season (226.7 mm), soil water content was the dominant controlling factor on soil respiration during the plant growing season [46]. Because of the accumulation of precipitation during June–July (Fig 2), soil water content was the primary limiting factor for net primary productivity [34] and microbial activity [49]. Inorganic sources and soil microbial activity are physically displaced and released during the dry period because of large amounts of CO_2_ stored in the air spaces [50]. In the wet year of 2012 (511.7 mm), the increasing soil respiration under different land management may have occurred when the liberated carbon held in large pools of soil carbonates was pulsed by increasing precipitation [50, 51] and soil rewetting rapidly increased decomposition processes of readily available carbon accumulated during previous dry periods by enhancing microbial C available for soil organic matter [52]. Our results show that the interannual variation in soil respiration and seasonal precipitation demonstrate the same variation tendency (Figs 2 and 5) [53], the large rainfall (511.7 mm) is associated with higher soil respiration (2.17 μmol.m^−2^.s^−1^) in 2012, whereas low rainfall (226.7 mm) is associated with lower soil respiration (1.42 μmol.m^−2^.s^−1^) in 2011. In addition, adding water to the soil crust (2 mm) and to the crust and subsoil (50 mm) to mimic large precipitation events (> 50 mm), may lead to an increases in soil respiration as it increase microbial activity in the Kalahari Desert of southern Africa [54]. Our results show that large precipitation events, such as the 178.1-mm event in July, 2012, stimulate large, discrete pulses of soil respiration, supporting the findings of previous studies [55]. These results highlight the importance of precipitation amount and pattern in determining the seasonal and inter-annual variation patterns of soil respiration.

## Conclusions

Soil temperature, soil water content and plant community primary production (ANPP and BNPP) are important factors in regulating the changes in soil respiration of temperate grassland under different management. The higher soil temperature, soil water content and NPP under enclosure protection has higher soil respiration than that under grazing use. Soil respiration is reduced by grazing as the management reduced soil moisture and inhibited plant production. Compared with the grassland in grazing treatment, clipping significantly increase BNPP when water limitation is weak. Precipitation plays a vital role in regulating land management effects on soil respiration. These findings help to improve our understanding of soil respiration under different land management in semiarid grassland ecosystems.

## References

[1] Jia BR, Zhou GS. Integrated diurnal soil respiration model during growing season of a typical temperate steppe: Effects of temperature, soil water content and biomass production. Soil Biology & Biochemistry. 2009; 41:681–686. 10.1016/j.soilbio.2008.12.030

[2] Thomey ML, Collins SL, Vargas R, Johnson JE, Brown RF, Natvig DO, et al Effect of precipitation variability on net primary production and soil respiration in a Chihuahuan Desert grassland. Global Change Biology. 2011; 17:1505–1515. 10.1111/j.1365-2486.2010.02363.x

[3] Cox PM, Betts RA, Jones CD, Spall SA, Totterdell IJ. Acceleration of global warming due to carbon-cycle feedbacks in a coupled climate model. Nature. 2000; 408:184–187. 11089968 10.1038/35041539

[4] Saiz G, Byrne KA, Butterbach-Bahl K, Kiese R, Blujdea V, Farrell EP. Stand age-related effects on soil respiration in a first rotation Sitka spruce chronosequence in central Ireland. Global Change Biology. 2006; 12:1007–1020. 10.1111/j.1365-2486.2006.01145.x

[5] Wan S, Luo Y. Substrate regulation of soil respiration in a tallgrass prairie: Results of a clipping and shading experiment. Global Biogeochemical Cycles. 2003; 17: n/a-n/a. 10.1029/2002gb001971

[6] Xia J, Han Y, Zhang Z, Wan S. Effects of diurnal warming on soil respiration are not equal to the summed effects of day and night warming in a temperate steppe. Biogeosciences. 2009; 6:1361–1370.

[7] Hungate BA, Holland EA, Jackson RB, Chapin FS, Mooney HA, Field CB. The fate of carbon in grasslands under carbon dioxide enrichment. Nature. 1997; 388:576–579.

[8] Yan LM, Chen SP, Huang JH, Lin GH. Water regulated effects of photosynthetic substrate supply on soil respiration in a semiarid steppe. Global Change Biology. 2011; 17:1990–2001. 10.1111/j.1365-2486.2010.02365.x

[9] Hanson PJ, Edwards NT, Garten CT, Andrews JA. Separating root and soil microbial contributions to soil respiration: A review of methods and observations. Biogeochemistry. 2000; 48:115–146. 10.1023/A:1006244819642

[10] Cao GM, Tang YH, Mo WH, Wang YA, Li YN, Zhao XQ. Grazing intensity alters soil respiration in an alpine meadow on the Tibetan plateau. Soil Biology & Biochemistry. 2004; 36:237–243. 10.1016/j.soilbio.2003.09.010

[11] Bahn M, Knapp M, Garajova Z, Pfahringer N, Cernusca A. Root respiration in temperate mountain grasslands differing in land use. Global Change Biology. 2006; 12:995–1006. 10.1111/j.1365-2486.2006.01144.x

[12] Yuste JC, Janssens IA, Carrara A, Ceulemans R. Annual Q(10) of soil respiration reflects plant phenological patterns as well as temperature sensitivity. Global Change Biology. 2004; 10:161–169. 10.1111/j.1529-8817.2003.00727.x

[13] Franzluebbers K, Franzluebbers AJ, Jawson MD. Environmental controls on soil and whole-ecosystem respiration from a tallgrass prairie. Soil Science Society of America Journal. 2002; 66:254–262.

[14] Liu XZ, Wan SQ, Su B, Hui DF, Luo YQ. Response of soil CO_2_ efflux to water manipulation in a tallgrass prairie ecosystem. Plant and Soil. 2002; 240: 213–223. 10.1023/A:1015744126533

[15] Rustad LE, Campbell JL, Marion GM, Norby RJ, Mitchell MJ, Hartley AE, et al A meta-analysis of the response of soil respiration, net nitrogen mineralization, and aboveground plant growth to experimental ecosystem warming. Oecologia, 2001; 126:543–562. 28547240 10.1007/s004420000544

[16] Frank AB, Liebig MA, Hanson JD. Soil carbon dioxide fluxes in northern semiarid grasslands. Soil Biology & Biochemistry. 2002; 34: 1235–1241. Pii 10.1016/S0038-0717(02)00062-7

[17] LeCain DR, Morgan JA, Schuman GE, Reeder JD, Hart RH. Carbon exchange and species composition of grazed pastures and exclosures in the shortgrass steppe of Colorado. Agriculture, Ecosystems and Environment. 2002; 93: 421–435.

[18] Xu X, Shi Z, Li D, Zhou X, Sherry RA, Luo Y. Plant community structure regulates responses of prairie soil respiration to decadal experimental warming. Global Change Biology. 2015; 10.1111/gcb.12940 25846478

[19] Wilsey BJ, Parent G, Roulet NT, Moore TR, Potvin C. Tropical pasture carbon cycling, relationships between C source/sink strength, above-ground biomass and grazing. Ecology Letters. 2002; 5: 367–376.

[20] Lal R. The physical quality of soil on grazing lands and its effect on sequestering carbon In: Follet R.F., Kimble J.M., Lal R. (Eds.), The Potential of U.S. Grazing Lands to Sequester Carbon and Mitigate the Greenhouse Effect. 2001; Lewis Publishers, New York, pp. 249–266.

[21] Xu X, Niu S, Sherry RA, Zhou X, Zhou J, Luo Y. Interannual variability in responses of belowground net primary productivity (NPP) and NPP partitioning to long-term warming and clipping in a tallgrass prairie. Global Change Biology. 2012; 18: 1648–1656.

[22] Craine FM, Wedin DA. Determinants of growing season soil CO_2_ flux in a Minnesota grassland. Biogeochemistry. 2002; 59: 303–313.

[23] Zhou X, Wan S, Luo Y. Source components and interannual variability of soil CO_2_ efflux under experimental warming and clipping in a grassland ecosystem. Global Change Biology. 2007; 13: 761–775.

[24] Jia X, Shao M, Wei X. Responses of soil respiration to N addition, burning and clipping in temperate semiarid grassland in northern China. Agricultural and Forest Meteorology. 2012; 166–167: 32–40.

[25] Chen Z, Wang S. Typical Steppe Ecosystems of China. Science Press, Beijing, China 2000.

[26] IUSS Working Group WRB. World reference base for soil resources 2006, 2 edn. World soil resources reports FAO, Rome 2006.

[27] Hoffmann C, Funk R, Wieland R, Li Y, Sommer M. Effects of grazing and topography on dust flux and deposition in the Xilingele grassland, Inner Mongolia. Journal of Arid Environment. 2008; 72: 792–807.

[28] Steingrobe B, Schmid H, Claassen N. The use of the ingrowth core method for measuring root production of arable crops–influence of soil conditions inside the ingrowth core on root growth. Journal of Plant Nutrition and Soil Science. 2000; 163:617–622.

[29] Byrne BM. Structural equation modeling with AMOS: Basic concepts, applications, and programming: Routledge; 2013.

[30] Li GY, Sun SC. Plant clipping may cause overestimation of soil respiration in a Tibetan alpine meadow, southwest China. Ecological Research. 2011; 26:497–504. 10.1007/s11284-011-0806-7

[31] Carbone F, Yang DS, Giannini E, Zewail AH. Direct role of structural dynamics in electron-lattice coupling of superconducting cuprates. Proceedings of the National Academy of Sciences. 2008; 105:20161–20166. 10.1073/pnas.0811335106 PMC2629268 19095796

[32] Xu WH, Wan SQ. Water- and plant-mediated responses of soil respiration to topography, fire, and nitrogen fertilization in a semiarid grassland in northern China. Soil Biology & Biochemistry. 2008; 40:679–687. 10.1016/j.soilbio.2007.10.003

[33] Chapin PS, Bloom AJ, Field CB, Waring RH. Plant responses to multiple environmental factors. Bioscience. 1987; 37: 49–57.

[34] Liu WX, Xu WH, Han Y, Wang CH, Wan SQ. Responses of microbial biomass and respiration of soil to topography, burning, and nitrogen fertilization in a temperate steppe.Biology and fertility of soils. 2007; 44: 259–268. 10.1007/s00374-007-0198-6

[35] Metcalfe DB, Fisher RA, Wardle DA. Plant communities as drivers of soil respiration: pathways, mechanisms, and significance for global change. Biogeosciences. 2011; 8: 2047–2061.

[36] Lü X, Freschet GT, Flynn DFB, Han X. Plasticity in leaf and stem nutrient resorption proficiency potentially reinforces plant–soil feedbacks and microscale heterogeneity in a semi-arid grassland. Journal of Ecology, 2012;100: 144–150.

[37] Lü X, Fresechet GT, Kazakou E, Wang Z, Zhou S, Han X. Contrasting responses in leaf nutrient-use strategies of two dominant grass species along a 30-yr temperate steppe grazing exclusion chronosequence. Plant and Soil. 2015; 387: 69–79.

[38] González D, Seastedt TR. Soil fauna and plant litter decomposition in tropical and subalpine forests. Ecology. 2001; 82: 955–964.

[39] de Deyn GB, Cornelissen H, Bardgett RD. Plant traits and soil carbon sequestration in contrasting biomes. Ecology Letters. 2008; 11: 516–531. 10.1111/j.1461-0248.2008.01164.x 18279352

[40] Lin Y, Hong M, Han GD, Zhao ML, Bai YF, Chang SX. Grazing intensity affected spatial patterns of vegetation and soil fertility in a desert steppe. Agriculture, Ecosystems & Environment. 2010; 138: 282–292.

[41] Maestre FT, Cortina J. Small-scale spatial variation in soil CO_2_ efflux in a Mediterranean semi-arid steppe. Applied Soil Ecology. 2003; 23: 199–209.

[42] Aguilar R, Ashworth L, Galetto L, Marcelo Adrián A. Plant reproductive susceptibility to habitat fragmentation: review and synthesis through a metaanalysis. Ecology Letters. 2006; 9: 968–980. 16913941 10.1111/j.1461-0248.2006.00927.x

[43] Joshi J, Stoll P, Rusterholz HP, Schmid B, Dolt C, Baur B. Small-scale experimental habitat fragmentation reduces colonization rates in species-rich grasslands. Oecologia. 2006; 148: 144–152. 16429312 10.1007/s00442-005-0341-8

[44] Lin XU, Zhang ZH, Wang SP, Hu YG, Xu GP, Luo CY, et al Response of ecosystem respiration to warming and grazing during the growing seasons in the alpine meadow on the Tibetan plateau. Agricultural and Forest Meteorology. 2011; 151: 792–802.

[45] Wang W, Fang JY. Soil respiration and human effects on global grasslands. Global Planet Change. 2009; 67:20–28. 10.1016/j.gloplacha.2008.12.011

[46] Yang YS, Chen GS, Guo JF, Xie JS, Wang XG. Soil respiration and carbon balance in a subtropical native forest and two managed plantations. Plant Ecology. 2007; 193: 71–84. 10.1007/s11258-006-9249-6

[47] Chen SP, Lin GH, Huang JH, Jenerette GD. Dependence of carbon sequestration on the differential responses of ecosystem photosynthesis and respiration to rain pulses in a semiarid steppe. Global Change Biology. 2009; 15: 2450–2461. 10.1111/j.1365-2486.2009.01879.x

[48] Wang CK, Yang JY, Zhang QZ. Soil respiration in six temperate forests in China. Global Change Biology. 2006; 12: 2103–2114. 10.1111/j.1365-2486.2006.01234.x

[49] Huxman TE, Snyder KA, Tissue D, Leffler AJ, Ogle K, Pockman WT, et al Precipitation pulses and carbon fluxes in semiarid and arid ecosystems. Oecologia. 2004; 141: 254–268. 15338414 10.1007/s00442-004-1682-4

[50] Emmerich WE. Carbon dioxide fluxes in a semiarid environment with high carbonate soils. Agricultural And Forest Meteorology. 2003; 116: 91–102.

[51] Inglima I, Alberti G, Bertolini T, Vaccari FP, Gioli B, Miglietta F, et al Precipitation pulses enhance respiration of Mediterranean ecosystems: the balance between organic and inorganic components of increased soil CO_2_ efflux. Global Change Biology. 2009; 15: 1289–1301.

[52] Gallo ME, Porras-Alfaro A, Odenbach KJ, Sinsabaugh RL. Photoacceleration of plant litter decomposition in an arid environment. Soil Biology & Biochemistry. 2009; 41: 1433–1441.

[53] Knapp AK, Briggs JM, Collins SL, Archer SR, Bret-Harte MS, Ewers BE, et al Shrub encroachment in North American grasslands: shifts in growth form dominance rapidly alters control of ecosystem carbon inputs. Global Change Biology. 2008; 14:615–623. 10.1111/j.1365-2486.2007.01512.x

[54] Thomas AD, Hoon SR, Dougill AJ. Soil respiration at five sites along the Kalahari Transect: effects of temperature, precipitation pulses and biological soil crust cover. Geoderma. 2011; 167–168: 284–294.

[55] Scholes RJ, Monteiro PMS, Sabine C, Canadell JG. Systematic long-term observations of global carbon cycle. Trends in Ecology & Evolution. 2009; 24: 427–430. 19409653 10.1016/j.tree.2009.03.006

