# Correction: Soil Respiration in Semiarid Temperate Grasslands under Various Land Management

**DOI:** 10.1371/journal.pone.0151719

**Published:** 2016-03-10

**Authors:** Zhen Wang, Lei Ji, Xiangyang Hou, Michael P. Schellenberg

In the Funding section, the grant number from the funder Program of the National Natural Science Foundation of China is listed incorrectly. The correct grant number is: 41471198.

## References

[1] Wang Z, Ji L, Hou X, Schellenberg MP (2016) Soil Respiration in Semiarid Temperate Grasslands under Various Land Management. PLoS ONE 11(1): e0147987 doi:10.1371/journal.pone.0147987 26808376 10.1371/journal.pone.0147987 PMC4726607

